# Association between a Genetic Risk Score Based on Single Nucleotide Polymorphisms of Coronary Artery Disease-Related Genes and Left Main Coronary Artery Disease

**DOI:** 10.1155/2018/8610368

**Published:** 2018-12-24

**Authors:** Zhengxi Xu, Hanning Liu, Cheng Sun, Ke Si, Yan Zhao, Zhe Zheng

**Affiliations:** ^1^State Key Laboratory of Cardiovascular Disease, Fuwai Hospital, National Center for Cardiovascular Diseases, Chinese Academy of Medical Sciences and Peking Union Medical College, Beijing, China; ^2^The Second Affiliated Hospital of Kunming Medical University, Kunming, China

## Abstract

Coronary artery disease (CAD) is the leading cause of mortality and morbidity worldwide. Left main coronary artery disease (LMCAD) is a severe phenotype of CAD and has a genetic component. Previous studies identified 3 inflammation-related single nucleotide polymorphisms (SNPs) contributing to the development of LMCAD. We integrated these SNPs into a genetic risk score for the prediction of LMCAD. We enrolled 1544 patients with CAD between 2007 and 2011. The individual associations of the 3 SNPs with LMCAD were assessed. We then calculated the genetic risk score for each patient and stratified patients into low-risk, intermediate-risk, and high-risk categories of genetic risk. In univariable logistic regression analysis, the odds of LMCAD for the high-risk group were 2.81 (95% confidence interval [CI]: 1.72-4.60; *P* = 0.02) times those of the low-risk group. After adjustment for CAD-related clinical variables, the high-risk group (adjusted OR: 2.78; 95% CI: 1.69-4.58; *P* = 0.02) had increased odds of LMCAD when compared with the low-risk group. Comparison of model c-statistics showed greater predictive value with regard to LMCAD for the genetic risk score model than the models including single SNPs.

## 1. Introduction

Among the various anatomic types of coronary artery disease (CAD), significant left main coronary artery disease (LMCAD) is the highest-risk lesion subset and is associated with poor clinical outcomes [1]. The left main coronary artery (LMCA), which arises from the left aortic sinus, provides more than 75% of the blood supply to the left ventricle. As a result, lesions in the LMCA can result in life-threatening events [2, 3].

Previous studies have confirmed the high heritability of LMCAD, showing asymptomatic siblings of patients with LMCAD have increased risk of future cardiovascular events [4, 5]. Recent research into the inflammatory nature of atherosclerosis has led to an improved mechanistic understanding of its pathogenesis, suggesting that inflammation plays an important role in the initiation, progression, and clinical outcomes of CAD and other manifestations of atherosclerosis. Blood-borne inflammatory and immune cells constitute an important part of atherosclerotic lesions, and many of the immune cells exhibit signs of activation and produce inflammatory cytokines [6, 7]. Furthermore, systemic inflammatory rheumatic diseases are associated with an increased risk of atherosclerotic events and premature cardiovascular disease [8].

With the greater availability of genetic testing, its use for estimating disease risk and characterizing patients with higher disease risk is of increasing scientific and public health interest [9, 10]. Based on the heritability and the pathogenesis of LMCAD, our previous studies identified several genetic variants of inflammation-related genes in patients with CAD that could contribute to the risk of LMCAD. Our results indicated that the single nucleotide polymorphisms (SNPs) interleukin-6 receptor (IL-6R) rs7529229 T/C [11] and hepatocyte nuclear factor 1 alpha (HNF1A) rs7310409 G/A [12] play essential roles in the inflammatory reaction and initiation process of CAD, thus contributing to the risk of LMCAD. Furthermore, the cyclooxygenase-2 (COX-2) rs5277 G/C polymorphism was associated with LMCAD, and rs5277 C allele carrier status was associated with the clinical outcomes of coronary artery bypass grafting among patients diagnosed with LMCAD [13].

The individual SNPs identified in our studies had only subtle effects on the risk of LMCAD, and the clinical use of these genetic variants for the prediction of LMCAD risk was limited. Accordingly, this study had 2 goals: (1) to establish a genetic risk score model based on the 3 SNPs and (2) to assess whether LMCAD was more likely among those at higher genetic risk compared to those at lower genetic risk according to the genetic risk score.

## 2. Materials and Methods

### 2.1. Study Subjects

The study protocol was approved by the Ethics Committee of Fuwai Hospital (Beijing, China), and we strictly complied with the World Medical Association Declaration of Helsinki. All patients provided written informed consent for their participation.

We recruited 1544 patients with CAD from the Cardiovascular Institute and Fuwai Hospital, Chinese Academy of Medical Sciences, and Peking Union Medical College (Beijing, China) between December 2007 and December 2011. All patients were genetically unrelated ethnic Han Chinese, and their CAD diagnosis was made by angiography and confirmed by surgery. LMCAD was defined as a lesion compromising the lumen by >50%, proximal to the bifurcation, including ostial stenosis. Lesions compromising the lumen by >50% outside of the LMCA were defined as more peripheral coronary artery disease (MPCAD) [14]. Clinical research staff collected baseline data for participating patients and double-entered the data into the study database.

### 2.2. DNA Isolation and Genotyping

Blood sample collection and genomic DNA isolation and genotyping of candidate SNPs were conducted as previously described [11–13]. Briefly, nurses collected blood samples into vacuum tubes containing ethylenediamine tetra-acetic acid. The genomic DNA was then isolated from whole blood using the Wizard Genomic DNA Purification Kit (Promega, Madison, WI, USA). DNA sample quality was assessed by performing polymerase chain reaction on the samples and visually analyzing the results on a 3% agarose gel with ethidium bromide staining. Genotyping of the SNPs (IL-6R rs7529229 T/C, HNF1A rs7310409 G/A, and COX-2 rs5277 G/C) was performed by matrix-assisted laser desorption ionization time-of-flight (MALDI-TOF) mass spectrometry with support from CapitalBio Corporation (Beijing, China). Matrix-assisted laser desorption ionization time-of-flight mass spectrometry (MALDI-TOF MS), the simplest and lowest-resolution form of mass spectrometry, has enhanced our ability to identify different genotype of different cell. The identification of different mammalian cell types using MALDI-TOF MS was first carried out by Zhang et al. [15]. Samples for MALDI analysis need to be cocrystallized with a large molar excess of matrix (usually a UV-absorbing organic acid) on target plates. The sample ions are then generated by laser radiation, followed by desorption and ionization processes. The matrix plays a key role because it absorbs the laser light energy and indirectly causes the analyte to vaporize. A time-of-flight (TOF) mass analyzer measures the mass dependent time required for ions of different masses to move from the ion source to the detector; the masses of the ions are determined from the time it takes to travel this distance. TOF is the fastest MS analyzer available, and it is well suited for pulsed ionization methods such as MALDI [16]. In our study, samples were transferred to a 384-well SpectroCHIP (Sequenom, San Diego, CA, USA) using a MassARRAY Nanodispenser (Sequenom) and analyzed by MALDI-TOF mass spectrometry. MassARRAY RT genotype-calling software (version 3.1; Sequenom) was used to call each genotype in real time.

### 2.3. Genetic Risk Score

The genetic risk score model was created from the SNPs identified in our prior studies (IL-6R rs7529229 T/C, HNF1A rs7310409 G/A, and COX-2 rs5277 G/C), which were all significantly associated with LMCAD. The genetic risk score for each patient was constructed by summing the number of risk alleles (0, 1, or 2) for each of the 3 SNPs. For example, for the SNP IL-6R rs7529229 T/C, C is the risk allele, and the genotypes TT, TC, and CC were valued at 0, 1, and 2, respectively.

### 2.4. Statistical Analysis

All continuous variables are presented as means ± standard deviations, and categorical variables are presented as percentages. Differences in demographic characteristics, clinical variables, and genotypes of the 3 SNPs by CAD type were evaluated using Student's *t* tests for continuous variables and chi-squared tests for discrete variables. The individual associations of the 3 SNPs with the LMCAD phenotype were estimated by computing odds ratios (ORs) and 95% confidence intervals (CIs) in univariable and multivariable logistic regression analyses. Age, sex, body mass index (BMI), smoking status, hypertension, hyperlipidemia, diabetes mellitus (DM), chronic renal dysfunction, chronic obstructive pulmonary disease (COPD), peripheral arterial disease (PAD), prior myocardial infarction (MI), prior percutaneous coronary intervention (PCI), and ejection fraction (EF) were included as covariates in the adjusted models. After calculating the genetic risk score for each patient in the study, univariable and multivariable logistic regression analyses were used to assess the association between the genetic risk score and the LMCAD phenotype. The C-statistic was calculated to evaluate the predictive value of this genetic risk score model. All statistical analyses were performed using SPSS software (version 19.0; SPSS Inc., Chicago, IL, USA), with 2-tailed tests for statistical significance and *α* = 0.05.

## 3. Results

### 3.1. Characteristics of the Study Population

There were 488 (31.6%) patients diagnosed with LMCAD by coronary angiography and 1056 (68.4%) patients diagnosed with MPCAD. The demographic and clinical characteristics of the patients are presented in Table 1. Patients with LMCAD versus MPCAD were older (62.28 ± 8.47 years versus 60.88 ± 8.68 years; *P* = 0.003) and more likely men (83.4% versus 78.6%; *P* = 0.03). There were no significant differences between the two CAD groups in BMI, smoking, hypertension, hyperlipidemia, DM, chronic renal dysfunction, COPD, PAD, prior MI, prior PCI, or mean EF.

### 3.2. Association between CAD-Related SNPs and LMCAD

Genotyping success rates were 98.7% for rs7529229, 99.9% for rs7310409, and 99.9% for rs5277. Information on the 3 SNPs and the frequencies of each genotype are shown in Table 2. For rs7529229 T/C, the frequencies were 34.4% (TT), 49.3% (TC), and 16.4% (CC) in the LMCAD patient group and 38.1% (TT), 46.9% (TC), and 15.1% (CC) in the MPCAD group. For rs7310409 G/A, the frequencies were 27.1% (GG), 49.7% (GA), and 23.2% (AA) in the LMCAD group and 34.2% (GG), 45.8% (GA), and 20% (AA) in the MPCAD group. For rs5277 G/C, the frequencies were 88.7% (GG), 11.3% (GC), and 0% (CC) in the LMCAD group and 92.5% (GG), 7.4% (GC), and 0.1% (CC) in the MPCAD group.

As shown in Table 3, there was no significant relationship between rs7529229 and LMCAD in either univariable or multivariable logistic regression analysis. For rs7310409, the odds of LMCAD for patients carrying one risk allele rs7310409 A were 1.22 (95% CI: 1.05-1.42; *P* = 0.009) times those of patients without this risk allele. For rs5277, the odds of LMCAD for patients carrying one risk allele rs5277 C were 1.54 (95% CI: 1.08-2.21; *P* = 0.02) times those of patients without this risk allele. After adjustment for age, sex, and clinical characteristics, carriers of the 7310409 A allele (adjusted OR: 1.23; 95% CI: 1.06-1.43; *P* = 0.007) and the rs5277 C allele (adjusted OR: 1.56; 95% CI: 1.09-2.24; *P* = 0.02) continued to have greater odds of LMCAD.

### 3.3. Genetic Risk Score for the Prediction of LMCAD

**Figure 1:**
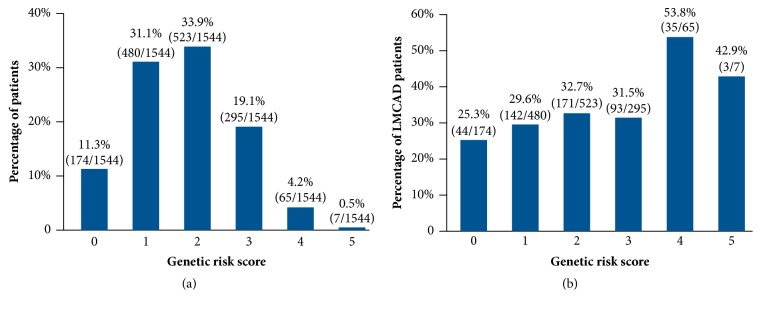
(a) Genetic risk score distribution in the study cohort and (b) percentage of LMCAD patients in each score category.

The genetic risk score was 0 for 174 (11.3%) patients, 1 for 480 (31.1%) patients, 2 for 523 (33.9%) patients, 3 for 295 (19.1%) patients, 4 for 65 (4.2%) patients, and 5 for 7 (0.5%) patients (Figure 1(a)). The percentages of patients with LMCAD in these increasing score categories were 25.3%, 29.6%, 32.7%, 31.5%, 53.8%, and 42.9%, respectively (Figure 1(b)). After grouping patients into 3 categories based on the genetic risk score, there was a significant increased odds of LMCAD for those in the high-risk group compared with the low-risk group (unadjusted OR: 2.81; 95% CI: 1.72-4.60; *P* = 0.02). In analyses adjusted for age, sex, and clinical characteristics, the odds of LMCAD were greater for both the intermediate-risk (adjusted OR: 1.23; 95% CI: 0.98-1.54; *P* = 0.007) and high-risk (adjusted OR: 2.78; 95% CI: 1.69-4.58; *P* = 0.02) groups when compared with the low-risk group (Table 4). Power calculations for our analysis are shown in Table 5. We had more than 99% power to detect an OR of 1.8, 64.4% power to detect an OR of 1.30.

### 3.4. Predictive Value of the Genetic Risk Score versus Single SNP

The C-statistics from the individual SNP logistic regression models were 0.521 (95% CI: 0.490-0.552; *P* = 0.19) for rs7529229, 0.541 (95% CI: 0.510-0.572; *P* = 0.01) for rs7310409, and 0.520 (95% CI: 0.488-0.551; *P* = 0.21) for rs5277. The genetic risk score model had a C-statistic of 0.547 (95% CI: 0.516-0.578; *P* = 0.003) (Table 6).

## 4. Discussion

In this study of more than 1500 patients with CAD, a genetic risk score was created from SNPs of 3 inflammation-related genes (IL-6R rs7529229 T/C, HNF1A rs7310409 G/A, and COX-2 rs5277 G/C) and all these genes are reported to be involved in the pathogenesis of CAD. The genetic risk score for each patient was constructed by summing the number of risk alleles (0, 1, or 2) for each of the 3 SNPs. The individual SNPs were associated with increased odds of LMCAD, though the findings for rs7529229 did not reach statistical significance. We found that in this cohort, the percentage of LMCAD is higher in patients with higher genetic risk score and the genetic risk score was significantly associated with LMCAD, indicating that patients with a higher genetic risk score may be more predisposed to the development of LMCAD. The C-statistic for this genetic risk score model was higher than that for the individual SNP models, indicating that integration of 3 SNPs into a single genetic risk score could provide an improved predictive tool for LMCAD. Such a tool could be particularly helpful in the clinical setting.

The LMCA refers to the proximal segment of the left coronary artery arising from the left aortic sinus just below the sinotubular junction to its bifurcation into the left anterior descending and left circumflex arteries. Severe LMCAD will reduce blood flow to a large portion of the myocardium, placing the heart at high risk for life-threatening left ventricular dysfunction and arrhythmias [3]. The severity and high prevalence of LMCAD have encouraged researchers to investigate a variety of approaches to early and accurate recognition of the disease. Previously, there was no SNP-based genetic risk score for the prediction of LMCAD risk. However, our study suggests that such a score might predict LMCAD. Our prior work showed that IL-6R rs7529229 T/C (adjusted OR: 1.31; 95% CI: 1.02-1.69; *P* =.04) [11], HNF1A rs7310409 G/A (adjusted OR: 1.45; 95% CI: 1.03-2.04; *P* =.03) [12], and COX-2 rs5277 G/C (adjusted OR: 1.59; 95% CI: 1.10-2.29; *P* =.01) [13] polymorphisms contribute to the risk of LMCAD. Furthermore, the 3 genes are strongly associated with the pathogenesis and adverse effects of CAD. As an essential cytokine, IL-6 has a broad range of immune properties relating to inflammation and tissue injury, and it contributes to the clinical evolution of CAD [17, 18]; it exerts its biological activities through IL-6R. HNF1A encodes a transcription factor that is expressed in many different tissues (e.g., liver, alimentary tract, kidney, and pancreas) and involved in the inflammation reaction of many disease processes via the promotion of C-reactive protein expression [19]. COX, also known as prostaglandin endoperoxide synthase, is a rate limiting enzyme that converts free arachidonic acid into important prostaglandins and eicosanoids such as prostaglandin H2 [20]. Two isoforms of COX, COX-1 and COX-2, have been identified within atherosclerotic lesions; COX-2 is dominantly expressed in macrophage and foam cells, which indicates crucial participation in the process of atherosclerosis [21]. Based on these physiological functions and our previous findings, we combined these 3 SNPs into a genetic risk score for LMCAD and found that stratifying CAD patients by genetic risk may identify a subset of adults who are more likely to have LMCAD.

Unlike our genetic risk score in this study, which was based solely on the genetic background of patients with CAD, more widely used clinical risk scores for the prediction of CAD are based on anatomical, clinical, biochemical, and imaging parameters. The widely cited Framingham risk score was constructed decades earlier and is used around the world [22]. The score represents a continuous scale estimate of the 10-year risk of a coronary heart disease event, and it is routinely used to categorize people into low-risk, intermediate-risk, and high-risk groups [23]. In clinical practice, those categorized as high risk (10-year risk: ≥20%) are often provided the most intensive treatment, and those at intermediate risk (10-year risk: 1%-20%) receive less intensive medical intervention [24]. Recently, researchers expanded the Framingham risk score to include other clinical parameters that predict CAD in different patient subgroups with higher accuracy [25–27].

The development and use of genetic risk scores has been limited in the past. However, genome-wide association studies and candidate gene-related research have identified a tremendous number of genetic variants in the process of specific diseases and pathophysiological disorders. The integration of susceptibility loci in a genetic risk score model with traditional clinical risk scoring systems could assist clinicians with the evaluation of disease risk or the determination of the optimal treatment strategy. Several genetic risk score models have been used in cardiovascular disease settings outside of LMCAD. One study found that incorporating a genetic risk score in coronary heart disease risk estimates could assist in the control of low-density lipoprotein cholesterol levels [28]. A meta-analysis of 48,421 individuals and 3477 events from a community-based cohort study (the Malmo Diet and Cancer Study) and 4 randomized controlled trials of statin therapy (JUPITER, ASCOT, CARE, and PROVE IT-TIMI 22) found an association between a polygenic risk score based on 27 genetic variants and the incidence and recurrence of coronary heart disease [10]. In the WOSCOPS trial, an expanded polygenic risk score with 57 SNPs was used to identify individuals at high genetic risk; statin therapy was associated with a reduction in the risk of CAD from 44% among those at high genetic risk versus 24% among all others. Additionally, this study demonstrated that patients at higher genetic risk had an increased burden of atherosclerosis in both coronary and carotid arteries [29]. In another study, a genetic QT score comprising 61 common genetic variants explained a significant proportion of the variability in drug-induced QT prolongation and was a significant predictor of drug-induced torsade de pointes [30].

Several potential limitations of the study should be considered. First, selection bias was unavoidable due to the hospital-based design. Second, the polymorphisms we investigated were based on functional considerations, and they may not offer a comprehensive view of the genetic variability underlying these phenotypes. Third, only 3 SNPs were chosen as candidates for the score development so the predictive value is not high; more genetic risk variants should be included to expand this genetic risk score and more accurately identify patients with increased LMCAD risk. Fourth, replication of our study in larger samples and more ethnically diverse populations is needed to confirm the findings.

In conclusion, this study provides evidence of an association between a genetic risk score and LMCAD. Compared with a single SNP as a predictive tool for LMCAD, the predictive value of this genetic risk score was higher, which might help to better identify a subset of patients at greater risk of LMCAD in clinical practice.

## Figures and Tables

**Table 1:** Baseline characteristics of patients.

	**LMCAD (n=488)**	**MPCAD (n=1056)**	**p value**
Age (yr)	62.28 (±8.47)	60.88 (±8.68)	**0.003**
Male Sex	407 (83.4)	830 (78.6)	**0.028**
BMI (kg/m^2^)	25.51 (±3.15)	25.87 (±5.51)	0.183
Smoking	248 (50.8)	536 (50.8)	0.982
Hypertension	320 (65.6)	706 (66.9)	0.620
Hyperlipidemia	329 (67.4)	713 (67.5)	0.969
Diabetes mellitus	154 (31.6)	360 (34.1)	0.326
Chronic renal dysfunction	2 (0.4)	9 (0.9)	0.519
COPD	2 (0.4)	5 (0.5)	1.000
Peripheral vascular disease	15 (3.1)	19 (1.8)	0.113
Prior MI	162 (33.2)	424 (40.2)	0.009
Prior PCI	48 (9.8)	102 (9.7)	0.913
EF (%)	60.20 (±8.07)	59.38 (±8.87)	0.071

Values are mean (±SD) or n (%). LMCAD indicates left main coronary artery disease; MPCAD: more peripheral coronary artery disease; BMI: body mass index; COPD: chronic obstructive pulmonary disease; MI: myocardial infarction; PCI: percutaneous coronary intervention; EF: ejection fraction.

**Table 2:** Primary information of genotyped SNPs.

**Genotyped SNP**	**Success rate**	**Genotype**	**Frequencies**	
			**LMCAD (**%**)**	**MPCAD (**%**)**
rs7529229	98.7%	TT	165 (34.4)	397 (38.1)
		TC	237 (49.3)	489 (46.9)
		CC	79 (16.4)	157 (15.1)
rs7310409	99.90%	GG	132 (27.1)	361 (34.2)
		GA	242 (49.7)	483 (45.8)
		AA	113 (23.2)	211 (20.0)
rs5277	99.90%	GG	432 (88.7)	977 (92.5)
		GC	55 (11.3)	78 (7.4)
		CC	0 (0)	1 (0.1)

SNP indicates single nucleotide polymorphism

**Table 3:** Main effects of SNPs on LMCAD risk.

**SNP**	**Unadjusted**			**Adjusted**		
	**OR**	**95**%** CI**	***p***	**OR**	**95**%** CI**	***p***
rs7529229	1.113	0.952~1.301	0.178	1.108	0.946~1.297	0.203
rs7310409	1.220	1.051~1.417	0.009	1.230	1.058~1.431	0.007
rs5277	1.542	1.077~2.207	0.018	1.560	1.087~2.239	0.016

**Table 4:** High genetic risk is associated with increased LMCAD risk.

	**Unadjusted**			**Adjusted**		
	**OR**	**95**%** CI**	***p***	**OR**	**95**%** CI**	***p***
Low-risk	-	-	1.85×10^−4^	-	-	2.37×10^−4^
Intermediate-risk	1.199	0.958~1.501	0.113	1.228	0.979~1.541	0.007
High-risk	2.812	1.718~4.604	0.018	2.781	1.688~4.581	0.016

**Table 5:** Power estimates for various relative risks (low-risk group vs. intermediate and high-risk group, two-sided alpha 0.05).

**RR**	**CAD cohort** **LMCAD 488 (31.6**%**),** **MPCAD 1056 (68.4**%**)**
1.3	0.644
1.5	0.947
1.8	0.999

**Table 6:** Predictive value of single SNP and genetic risk score for LMCAD risk.

**Genetic factor**	**C statistic**	**95% CI**	***p***
rs7529229	0.521	0.490~0.552	0.187
rs7310409	0.541	0.510~0.572	0.011
rs5277	0.520	0.488~0.551	0.214
Genetic risk score	0.547	0.516~0.578	0.003

## Data Availability

The data used to support the findings of this study are available from the corresponding author upon request.
